# Characterization of Mu-Like *Yersinia* Phages Exhibiting Temperature Dependent Infection

**DOI:** 10.1128/spectrum.00203-23

**Published:** 2023-07-19

**Authors:** Biao Meng, Zhizhen Qi, Xiang Li, Hong Peng, Shanzheng Bi, Xiao Wei, Yan Li, Qi Zhang, Xiaoqing Xu, Haihong Zhao, Xiaoyan Yang, Changjun Wang, Xiangna Zhao

**Affiliations:** a Department of Epidemiology and Biostatistics, School of Public Health, Anhui Medical University, Hefei, China; b Institute of Disease Control and Prevention, Chinese PLA, Beijing, China; c Qinghai Institute for Endemic Disease Prevention and Control of Qinghai Province, Key Laboratory for Plague Prevention and Control of Qinghai Province, Xining, China; Institut Pasteur

**Keywords:** *Marmota himalayana*, *Yersinia pestis*, Mu-like phages, temperature dependent infection

## Abstract

Yersinia pestis is the etiological agent of plague. *Marmota himalayana* of the Qinghai-Tibetan plateau is the primary host of flea-borne Y. pestis. This study is the report of isolation of Mu-like bacteriophages of Y. pestis from *M. himalayana*. The isolation and characterization of four Mu-like phages of Y. pestis were reported, which were named as vB_YpM_3, vB_YpM_5, vB_YpM_6, and vB_YpM_23 according to their morphology. Comparative genome analysis revealed that vB_YpM_3, vB_YpM_5, vB_YpM_6, and vB_YpM_23 are phylogenetically closest to Escherichia coli phages Mu, D108 and Shigella flexneri phage SfMu. The role of LPS core structure of Y. pestis in the phages’ receptor was pinpointed. All the phages exhibit “temperature dependent infection,” which is independent of the growth temperature of the host bacteria and dependent of the temperature of phage infection. The phages lyse the host bacteria at 37°C, but enter the lysogenic cycle and become prophages in the chromosome of the host bacteria at 26°C.

**IMPORTANCE** Mu-like bacteriophages of Y. pestis were isolated from *M. himalayana* of the Qinghai-Tibetan plateau in China. These bacteriophages have a unique temperature dependent life cycle, follow a lytic cycle at the temperature of warm-blooded mammals (37°С), and enter the lysogenic cycle at the temperature of its flea-vector (26°С). A switch from the lysogenic to the lytic cycle occurred when lysogenic bacteria were incubated from lower temperature to higher temperature (initially incubating at 26°C and shifting to 37°C). It is speculated that the temperature dependent lifestyle of bacteriophages may affect the population dynamics and pathogenicity of Y. pestis.

## INTRODUCTION

Yersinia pestis, the causative agent of plague, is transmitted by flea bites or aerosols and causes highly severe infections, i.e., bubonic and pneumonic plague (1). *Marmota himalayana* is the main natural host of plague and is endemic to the Qinghai-Tibetan Plateau, China (2, 3). Fleas are important vectors of Y. pestis between the host (wild animals or synanthropic rodents) and humans (3). From 1995 to 2021, 7 Y. pestis isolates with four antibiotic resistance mechanisms were reported (4). Bacteriophages present huge potential both as a resource for developing novel tools for bacterial diagnostics and for use in phage therapy (5). Y. pestis phages have been used for diagnosis purposes (6, 7), and extensive research has focused on the genomic characterization (8), phage receptors identification (9), and biotechnological applications (10). In addition, applications of Y. pestis phages as therapeutic agents have been investigated (11). Since *M. himalayana* is a natural host of Y. pestis, it may represent an interesting source to isolate phages. However, limited information exists on phages from *M. himalayana* and on the impact of environmental conditions on susceptibility of Y. pestis strains to such phages. In this study, we collected *M. himalayana* that had died of natural causes from Ulan County of Haixi Mongolian and Tibetan Autonomous Prefecture of Qinghai province. Four phages were isolated from *M. himalayana* and the characteristics were identified. Based on the phylogenetic analysis of the genetic sequences, these phages were classified as the Mu-like bacteriophage of *Myoviridae*. Our previous observation of Y. pestis concerned the discovery of a P2-like *Yersinia* phage that exhibits “temperature dependent infection” (12). In this work, these Mu-like *Yersinia* phages also exhibit such phenotype, it was shown that these phages follow a lytic cycle in warm conditions (37°C), but at colder temperatures (26°C) no lysis occurs. The mechanism of these condition dependent phages during infection were investigated. We provide experimental evidence that the phages have a temperature dependent lifestyle, and it is speculated that this factor has the potential to impact on the population dynamics and the pathogenicity of Y. pestis. The discovery of such phages has profound implications for our understanding of ecology and pathogenesis of Y. pestis.

## RESULTS

### Isolation and characterization of phages.

**FIG 1:**
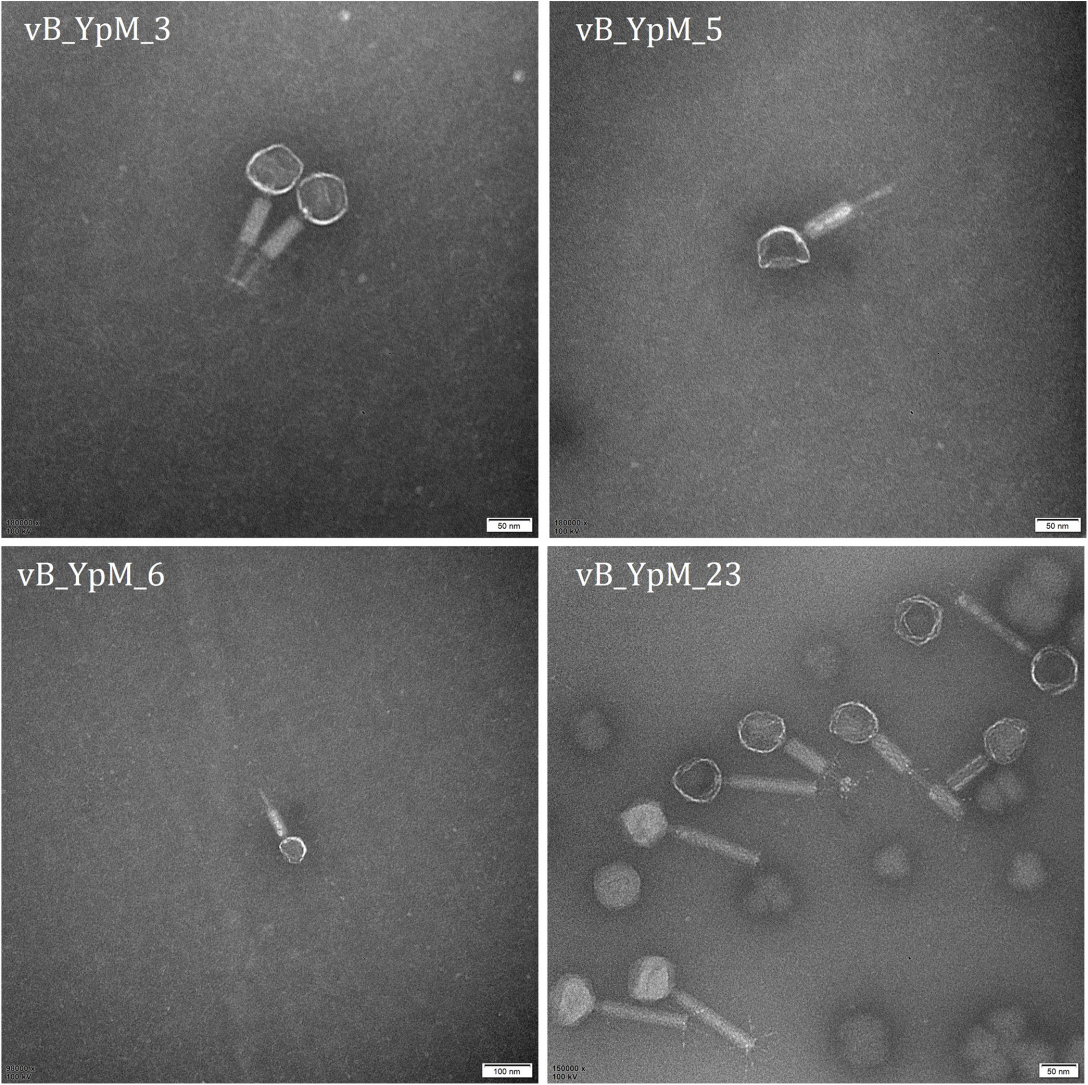
Electron micrograph of the phages, negatively stained with 2% phosphotungstenic acid.

To study Y. pestis-lysing phages present in *M. himalayana*, we sampled cecum from *M. himalayana* that had died of natural causes of Qinghai-Tibetan Plateau, China, and isolated four phages capable of infecting this bacterium. Y. pestis biovar Microtus strain 201 was used as an indicator and four phages named vB_YpM_3, vB_YpM_5, vB_YpM_6, and vB_YpM_23 were identified. Each of these phages could infect Y. pestis strain 201 culture forming small, transparent plaques on solidified plate (Fig. S1). All of these phages have icosahedral heads and contractile tails and were morphologically similar to the phages of family *Myoviridae* and order *Caudovirale* (13) (Fig. 1).

### Lytic activity.

**FIG 2:**
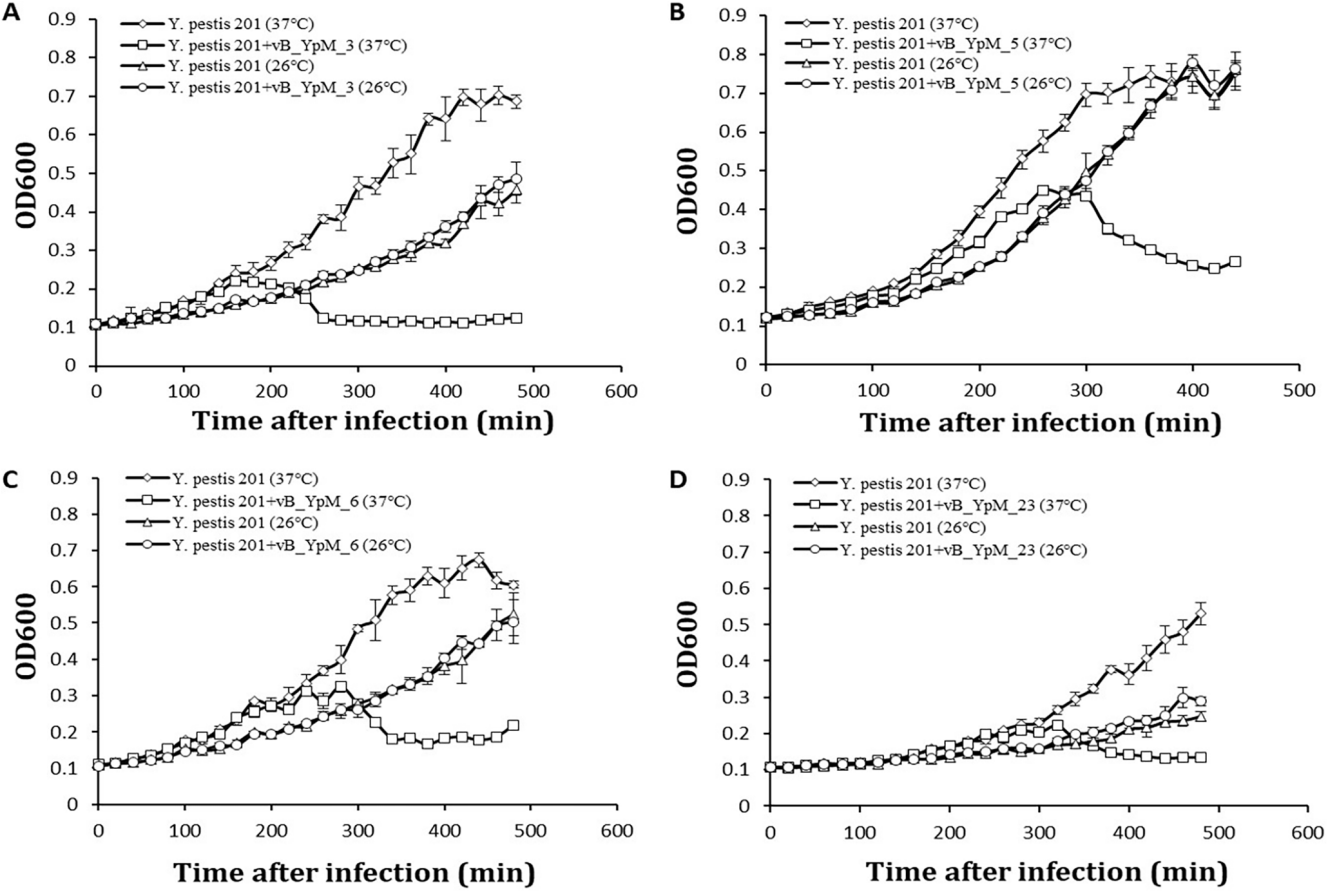
Infection assays of phages. Phages were grown in an exponential-phase culture of Y. pestis strain *201*. Each data point is a mean of three different replications of the same experiment.

The plaque-forming capacity of the phages was examined at 26°C and 37°C using 26 *Yersinia* strains. The phages are responsible for complete lysis of Y. pestis strains at 37°C but not 26°C. No significant signs of lysis were detected for most other *Yersinia* species. Two Y. enterocolitica strains, ATCC9610 and 52204, could be lysed by vB_YpM_6 and vB_YpM_23 at 37°C. One Y. pseudotuberculosis strain, number 1799, could be lysed by vB_YpM_3 and vB_YpM_23 at 37°C (Table 1). Furthermore, the susceptibility of 18 non-*Yersinia* strains to the phages’ infection was tested, and it was proved that the phages are specific for *Yersinia* species. As the infectivity of phages showed obvious temperature dependence, the liquid-based lysis test following the reduction in the OD of broth-suspended bacterial culture was compared for their ability to lyse Y. pestis at 26°C and 37°C (Fig. 2). As seen in Fig. 2, the phages showed lytic activity when the temperature of infection was 37°C but not when it was 26°C.

**TABLE 1:** Bacterial strains used for host range determination^*a*^

Bacteria strain	Description	vB_YpM_3		vB_YpM_5		vB_YpM_6		vB_YpM_23	
		26°C	37°C	26°C	37°C	26°C	37°C	26°C	37°C
Y. pestis 201(1)	biovar Microtus strain	−	+	−	+	−	+	−	+
Y. pestis EV76(2)	live attenuated vaccine strain	−	+	−	+	−	+	−	+
Y. enterocolitica ATCC9610(3)	standard ATCC strain	−	−	−	−	−	+	−	+
Y. enterocolitica 52204(3)	Ya96	−	−	−	−	−	+	−	+
Y. enterocolitica 52207(3)	Ya885	−	−	−	−	−	−	−	−
Y. pseudotuberculosis 3384(3)	serotype O1a, isolated from bird, Denmark	−	−	−	−	−	−	−	−
Y. pseudotuberculosis Pa3606(3)	serotype O1b, isolated from human, Japan	−	−	−	−	−	−	−	−
Y. pseudotuberculosis Kuratani(3)	serotype O1c, isolated from water, Japan	−	−	−	−	−	−	−	−
Y. pseudotuberculosis No.49(3)	serotype O2a, isolated from human, Japan	−	−	−	−	−	−	−	−
Y. pseudotuberculosis NO.1799(3)	serotype O2b, isolated from human, From Dr. Knapp	−	+	−	−	−	−	−	+
Y. pseudotuberculosis 274(3)	serotype O2c, isolated from pig, Japan	−	−	−	−	−	−	−	−
Y. pseudotuberculosis 83(3)	serotype O3, isolated from human, France	−	−	−	−	−	−	−	−
Y. pseudotuberculosis 51(3)	serotype O4a, isolated from pig, Japan	−	−	−	−	−	−	−	−
Y. pseudotuberculosis Pa3422(3)	serotype O4b, isolated from human, Japan	−	−	−	−	−	−	−	−
Y. pseudotuberculosis 204(3)	serotype O5a, isolated from human, Japan	−	−	−	−	−	−	−	−
Y. pseudotuberculosis 197(3)	serotype O5b, isolated from pig, Japan	−	−	−	−	−	−	−	−
Y. pseudotuberculosis DD110(3)	serotype O6, isolated from dog, Japan	−	−	−	−	−	−	−	−
Y. pseudotuberculosis 141(3)	serotype O7, isolated from mouse, Japan	−	−	−	−	−	−	−	−
Y. pseudotuberculosis 151(3)	serotype O8, isolated from pig, Japan	−	−	−	−	−	−	−	−
Y. pseudotuberculosis R708Lv(3)	R708Lv serotype O9, isolated from wild rat, Japan	−	−	−	−	−	−	−	−
Y. pseudotuberculosis 6088(3)	serotype O10, isolated from raccoon dog, Japan	−	−	−	−	−	−	−	−
Y. pseudotuberculosis R80(3)	serotype O11, isolated from wild rat, Japan	−	−	−	−	−	−	−	−
Y. pseudotuberculosis MW864-2(3)	serotype O12, isolated from mountain water, Japan	−	−	−	−	−	−	−	−
Y. pseudotuberculosis N916(3)	serotype O13, isolated from house rat, China	−	−	−	−	−	−	−	−
Y. pseudotuberculosis CN3(3)	serotype O14, isolated from wild rat, China	−	−	−	−	−	−	−	−
Y. pseudotuberculosis 93422(3)	serotype O15, isolated from human, South Korea	−	−	−	−	−	−	−	−
Escherichia coli ATCC 25922(4)	standard ATCC strain	−	−	−	−	−	−	−	−
E. coli EC600(5)	engineered E. coli strain	−	−	−	−	−	−	−	−
Klebsiella pneumoniae ATCC BAA-2146(6)	Standard ATCC strain	−	−	−	−	−	−	−	−
K. pneumoniae ATCC BAA-1705(7)	Standard ATCC strain	−	−	−	−	−	−	−	−
K. pneumoniae K2044(8)	clinical strain isolated from human, China	−	−	−	−	−	−	−	−
Staphylococcus aureus MU50(9)	clinical strain isolated from human, China	−	−	−	−	−	−	−	−
Serratia marcescens wk2050(3)	clinical strain isolated from human, China	−	−	−	−	−	−	−	−
Enterobacter aerogenes 3-SP(10)	clinical strain isolated from human, China	−	−	−	−	−	−	−	−
Achromobacter xylosoxidans A22732(11)	clinical strain isolated from human, China	−	−	−	−	−	−	−	−
E. cloacae T5282(12)	clinical strain isolated from human, China	−	−	−	−	−	−	−	−
*Leclercia adcarboxglata* P10164(13)	clinical strain isolated from human, China	−	−	−	−	−	−	−	−
Raoultella ornithinolytica YNKP001(14)	clinical strain isolated from human, China	−	−	−	−	−	−	−	−
Stenotrophomonas maltophilia 9665(12)	clinical strain isolated from human, China	−	−	−	−	−	−	−	−
Pseudomonas aeruginosa PA01(15)	clinical strain isolated from human, China	−	−	−	−	−	−	−	−
Shigella sonnei #1083(12)	clinical strain isolated from human, China	−	−	−	−	−	−	−	−
E. aerogenes 13208(12)	clinical strain isolated from human, China	−	−	−	−	−	−	−	−
S. marcescens 57(12)	clinical strain isolated from human, China	−	−	−	−	−	−	−	−
K. oxytoca 2014010901-7121(12)	clinical strain isolated from human, China	−	−	−	−	−	−	−	−

a −, Infection absent; +, Infection present.

### Receptor identification.

**FIG 3:**
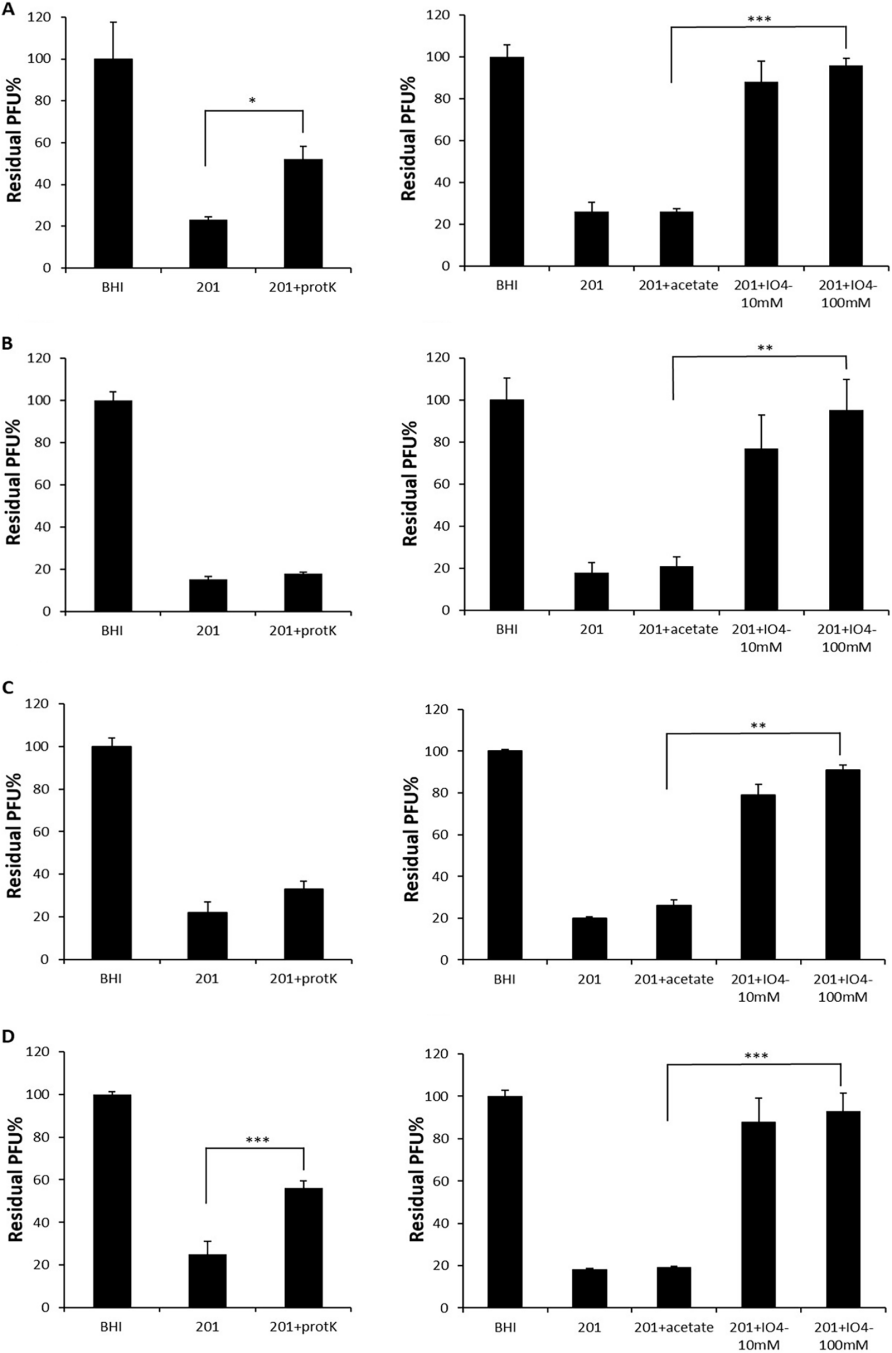
Effects of different treatments of bacteria on adsorption, shown as residual PFU percentages. (A) vB_YpM_3; (B) vB_YpM_5; (C) vB_YpM_6; (D) vB_YpM_23. Significance was determined by independent-sample *t* test for comparison. *, *P* < 0.05; ** *P*, < 0.01; ***, *P* < 0.001. BHI, the nonadsorbing control; 201, untreated strain; 201+ProtK, proteinase K treatment; 201+acetate, acetate buffer treatment; 201+10mM IO^4−^, 10 mM periodate treatment; 201+100mM IO^4−^, 100 mM periodate treatment. Each data point is a mean of three different replications of the same experiment.

**FIG 4:**
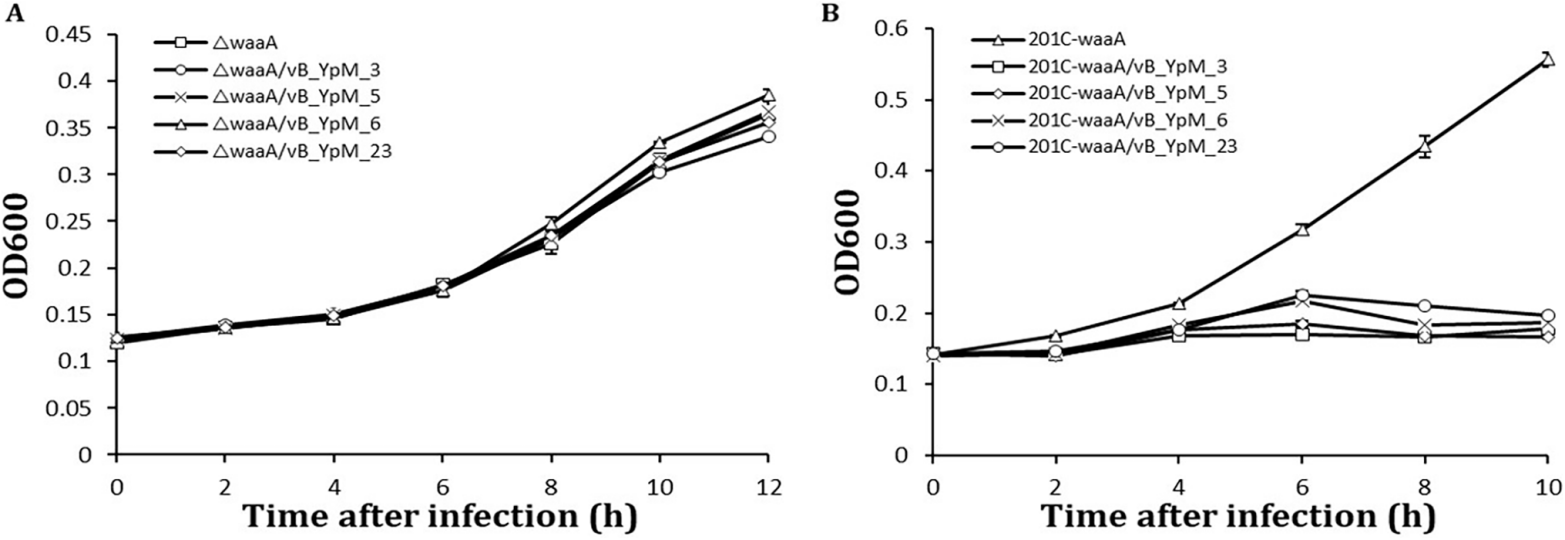
Infection assays of phages on the Δ*waaA* mutant strain and 201C-*waaA* strain. Each data point is a mean of three different replications of the same experiment.

Interaction of phage with bacterial cell is determined by specificity of adsorption, which is dependent on the nature and structural peculiarities of receptors on bacterial cell surface (14). Periodate can degrade carbohydrates containing a 1,2-diol motif in their structure (15), and proteinase K has a broad substrate specificity with its keratin hydrolyzing activity (16). To study how the degradation of cell surface proteins or carbohydrates affects the phage adsorption, an adsorption assay was conducted using Y. pestis cells treated with proteinase K or periodate. Incubation of Y. pestis in the presence of 100 mM periodate abolished these phages’ binding completely, whereas incubation in acetate buffer alone did not (Fig. 3). This indicated that a carbohydrate structure, most likely LPS, plays a critical role for the phages’ binding. The *waaA* gene encodes a Kdo transferase involved in the attachment of lipid-A to the core oligosaccharide, and a *waaA* mutant expresses only lipid A (17). To verify this possibility, a *waaA* mutant was used for the infection assay and the result proved that the *waaA* mutant was fully resistant to the infection of the phages (Fig. 4). Thus, the rough LPS core structure of Y. pestis as a critical part of the receptor for these phages was confirmed. Incubation of Y. pestis in the presence of proteinase K partially abolished the binding of phages vB_YpM_3 and vB_YpM_23 (Fig. 3). It is speculated that for phages vB_YpM_3 and vB_YpM_23, cell surface proteins of Y. pestis also involved in the adsorption of the phages.

### Bacteria-phage interactions.

**FIG 5:**
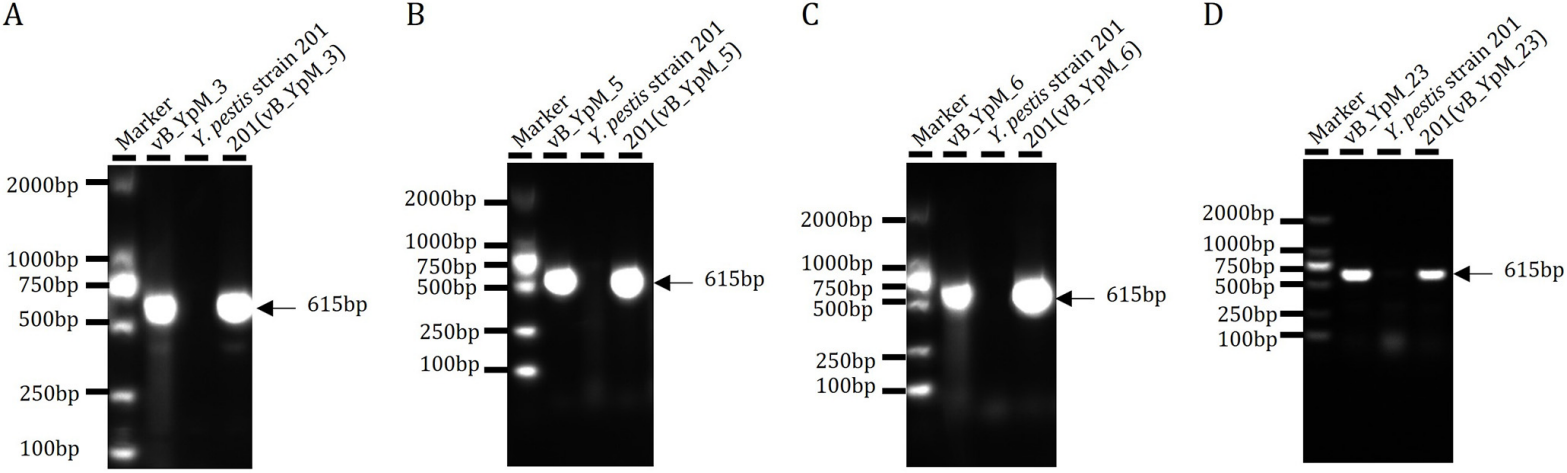
PCR results of the Y. pestis colonies using Mu-like phages specific primers, the colonies were originated from the Y. pestis culture infected by the phages at 26°C. 201(vB_YpM_3), the colony infected by phage vB_YpM_3; 201(vB_YpM_5), the colony infected by phage vB_YpM_5; 201(vB_YpM_6), the colony infected by phage vB_YpM_6; 201(vB_YpM_23), the colony infected by phage vB_YpM_23.

The Lytic curves indicated that the phages may be dependent on the temperatures of infection. As Y. pestis is transmitted by fleas from rodent reservoirs and has the flea/rodent life cycle. At the temperature of its flea-vector (26°С), Y. pestis expresses a profile of genes distinct from those expressed in a mammalian host (37°C) (18). It was initially speculated that the recognition sites (receptors) on Y. pestis cell surface did not exist at the lower temperature (26°С), resulting in insensitivity to bacteriophages. By adsorption assay, it was found that host bacteria cultured at different temperatures (26°C and 37°C) can be recognized and adsorbed by phages (Fig. S2), proving that bacteriophage receptors on the surface of host bacteria can be expressed normally at different temperatures (26°C and 37°C). Next, whether the phage susceptibility of Y. pestis strains is dependent on the growth temperature of Y. pestis was examined. Bacterial growth and the following phage infections were performed at 26°C and 37°C. Regardless of the temperature of growth of the bacteria, plaques were only obtained when the temperature of phage infection was 37°C but not 26°C. This indicates that Y. pestis susceptibility to the phage infection is dependent on the temperature of subsequent infection but not on the growth temperature of the host cells (Fig. S3). Previous study indicated that some phages lysogenize their bacterial hosts at colder temperature (19). To assess this possibility, Y. pestis was infected with the phages at 26°C, and then the bacteria were plated on LB agar and incubated at 26°C. Colonies were selected to determine the presence of prophages. The colonies that were phage positive using PCR were found (Fig. 5). When the PCR positive colonies were propagated at 37°C, free phage particles were obtained again (Fig. S4). This indicated that the temperature shift experiment with lysogens (initially incubating at 26°C and shifting to 37°C) caused a switch from the lysogenic to the lytic cycle.

### Genomic features.

**FIG 6:**
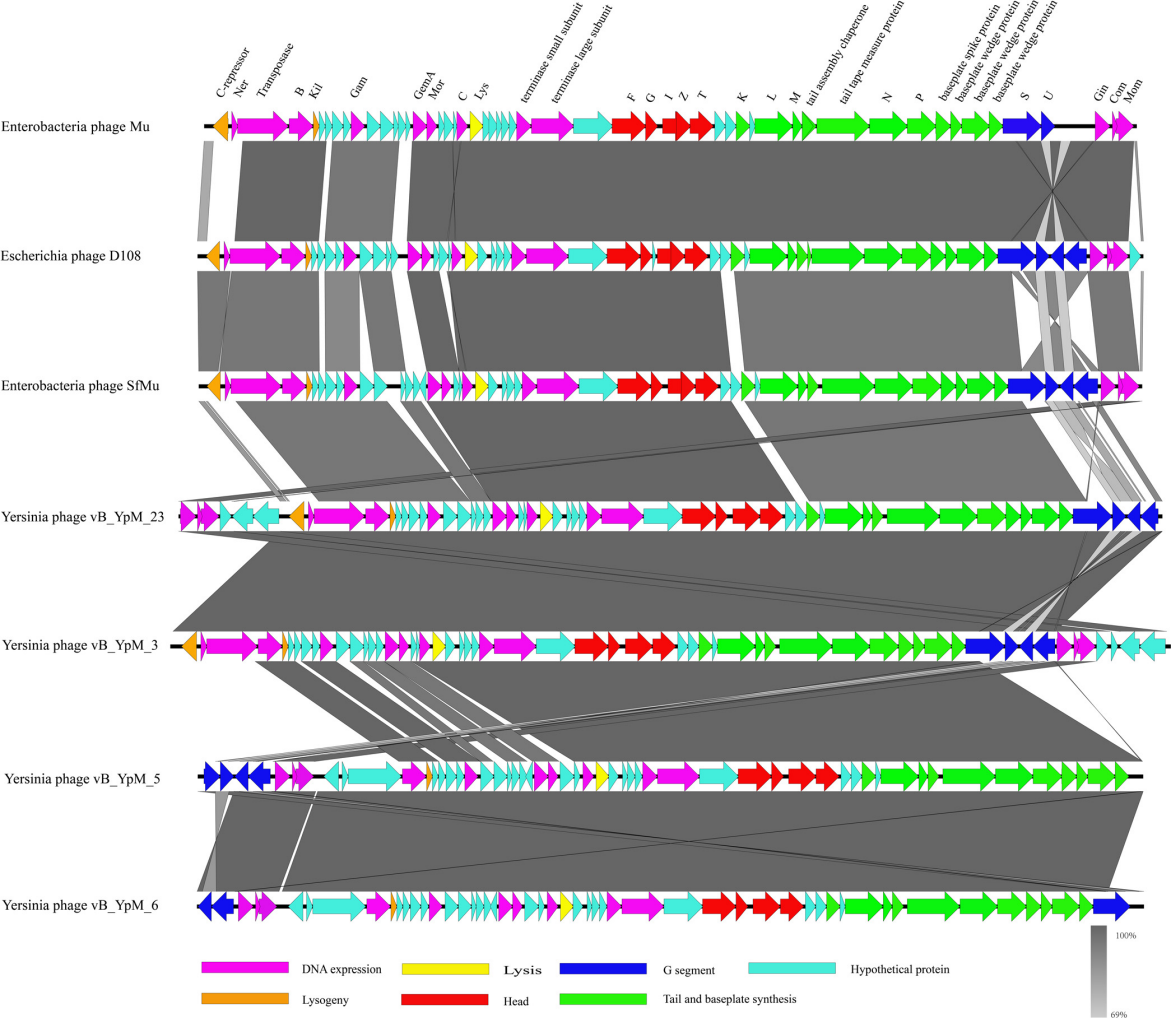
The comparative genomic analysis and the genetic map of vB_YpM_3 (MT374852), vB_YpM_5 (MT374853), vB_YpM_6 (MT374854), vB_YpM_23 (MT374856), Mu (AF083977), D108 (GQ357916), and SfMu (KP010268) based on blastn and Easyfig. Genes are indicated as arrows. Genes features are colored based on function classification.

Comparative genome analysis revealed that vB_YpM_3, vB_YpM_5, vB_YpM_6, and vB_YpM_23 belong to the family of Mu-like phages and are the close relative of E. coli phage Mu (AF083977), E. coli phage D108 (GQ357916), and Shigella flexneri bacteriophage SfMu (KP010268). The arrangement of genes in these phages was colinear with several differences (Fig. 6). It was divided into functional modules: DNA metabolism, lysis, structure (head assembly and tail assembly), etc. In these phages, the genes encoding structural proteins are highly conserved. The left end of genomes of these phages encodes the regulatory proteins, C-repressor and Ner, which are involved in the regulation of the lytic and lysogenic developments of the phages. C-repressor is required for the establishment and maintenance of lysogeny (20). Ner plays a central role in regulating the expression of the early (transposase) operon and in ensuring that phage growth proceeds along a lytic pathway (21). The invertible G segment includes genes S, U, U’, and S’, which are responsible for the tail fiber biosynthesis and assembly, and controls the host range of the phages (22). The G segment invertase (Gin) promotes inversion of the G segment and determines which pair of the genes is expressed: S and U or S’ and U’ (23). The frequency of the inversion reaction is low both in the lysogenic state and during lytic growth (24). Bacteria and phages have evolved DNA modification as a strategy to protect their genomes (25). DNA modification protein Mom protects the viral genome against a wide variety of restriction endonucleases (26). At the whole-genome level, vB_YpM_3 is closer to vB_YpM_23 and vB_YpM_5 is closer to vB_YpM_6 (Fig. S5).

## DISCUSSION

Plague is recognized as a reemerging disease and some antibiotic-resistant strains have been isolated from humans and rodents (11). As a renewed approach for the treatment of multidrug-resistant pathogens, attention was directed toward bacteriophages, viruses that infect bacteria. In this study, four Y. pestis-lysing phages from *M. himalayana* were characterized. Basic morphological and physiological properties of vB_YpM_3, vB_YpM_5, vB_YpM_6, and vB_YpM_23 have been described and they were identified as Mu-like phages. The genomes of the phages were highly comparable to that of Mu and its closest relative, phage D108 and SfMu, with conserved genomic arrangement. An understanding of the molecular mechanism of the specific adsorption of these phages is important for extending their applications (27). Previous study indicated that both bacteriophage Mu and D108 use LPS core as their receptors (22), and O-antigen serves as the receptor of phage SfMu (20). For all these Y. pestis-lysing phages, periodate treatment of Y. pestis inhibited the adsorption of these phages. In infection assays against a *waaA* mutant, we showed that if the gene, encoding the LPS-specific Kdo transferase, is mutated, the phages could not infect anymore. This suggested that LPS core of Y. pestis may play an important role in the adsorption of these phages. Proteinase K treatment of Y. pestis inhibited the adsorption of phages vB_YpM_3 and vB_YpM_23 to a certain degree, it is speculated that outer membrane proteins of Y. pestis also participate in the adsorption of vB_YpM_3 and vB_YpM_23. More surprising was the fact that these phages exhibited remarkable temperature dependency response when the temperature of infection altered. The phenomenon of temperature dependency in these phages is independent on the growth temperature of the bacteria host. They infected Y. pestis and killed the host cells at the temperature of warm-blooded mammals (37°С). Whereas at the temperature of poikilothermic fleas (26°С) they lysogenize Y. pestis. They have a temperature dependent life cycle at two environmentally relevant temperatures. It is highly plausible that these phages have a profound and hitherto unrecognized effect on the population dynamics of Y. pestis. It is likely that the phages have evolved to switch from a lysogenic to a lytic lifestyle when the temperature increased. When taken together with previous studies, our findings suggest that the phenomenon of temperature dependency in phages is not uncommon. Previous studies reported that in warm conditions the phages infect the pathogen and follow a lytic cycle (immediately killing the host cells), whereas at colder temperatures they lysogenize their bacterial hosts (28). It was speculated the phages entered the lytic state and lyse Y. pestis following the transition into a warm-blooded host, i.e., *M. himalayana*. And the phages entered a lysogenic lifestyle in hematophagous arthropod vectors, i.e., fleas. Most of the putative virulence factors of Y. pestis are transcriptionally regulated by temperature shifts and are active at 26°С or 37°С to obtain adaptability of growing in fleas or in warmblood mammalian hosts during its life cycle (29). For instance, the ure operon is transcriptionally upregulated at 26°С to likely reduce the toxicity to fleas, and thus maintaining an infection in a larger population (30). Temperature is likely to influence the dynamics of Y. pestis flea-borne transmission, perhaps by affecting persistence of Y. pestis in the flea gut (31). Whether and how these transposable temperate phages participate in this process is a critical issue. *Enterobacteria* phage Mu is the best studied transposable and temperate phage, and it could enter the lytic cycle or lysogenize its host by randomly integrating into the host chromosome (32). The life cycle switch of the Mu-like phages described in this study is temperature dependent based our data. However, the underlying mechanism is still unclear. It is necessary to compare the up- or downregulated genes of the lysogens and wild-type strains by RNA-Seq analyses and to find the determining factor of the life cycle switch. Due to the ability to integrate their genomes into the host chromosome, temperate phages are considered to affect the fitness and phenotype of host bacteria (33). There are still many unresolved issues; whether the integration of these phages increase or decrease the virulence of parental strain is worth further research. Phage integration generally occurs at a specific site in the host chromosome; it also occurs at other, so-called noncanonical sites or secondary sites (34). Further research is required to determine whether the transposable temperate phages form lysogens in Y. pestis via site-specific integration or off-site integration.

## MATERIALS AND METHODS

### Isolation and characterization of phages.

Phages were isolated from the cecum samples of *M. himalayana* as described previously (12). Four phages were isolated and selected for further analysis based on their ability to form clear plaques on a lawn of Y. pestis in the double-layer agar assay. Phages vB_YpM_3, vB_YpM_5, and vB_YpM_6 were isolated from *M. himalayana* (female) of 36°56′ in northern latitude and 98°43′ in eastern longitude at 3423.9 m. vB_YpM_23 was isolated from *M. himalayana* (male) of 36°55′ in northern latitude and 98°44′ in eastern longitude at 3557.2 m. Phage double-layer agar assays were performed using the PAMA technique as described previously (35). The host range of the phages was tested using two Y. pestis strains, three Y. enterocolitica strains, 21 Y. pseudotuberculosis strains, and 20 non-*Yersinia* strains as described previously (12). The *in vitro* infection assay of phages against Y. pestis at 26°C and 37°C was determined by optical densitometry (OD600) (36). Purified phage particles were stained with 2% potassium phosphotungstate (pH 7.0) and visualized using a Tecnai Spirit 120-kV transmission electron microscope (FEI Company, USA) for morphological identification. All experiments with Y. pestis strains were undertaken in the biosafety level 3 (BSL-3) laboratory at Qinghai Institute for Endemic Disease Prevention and Control of Qinghai Province.

### Genome sequencing and phylogenetic tree construction.

Phage DNA was purified using a method described in (37), followed by fragmentation of the DNA into approximately 350 bp fragments using a DNA fragmentation instrument (Covaris M220). Afterwards, we utilized the Illumina library construction kit for standard fragment library construction. The constructed library was subjected to fragment length analysis using Agilent 2100. After passing quality control, the library was sequenced with the Illumina NovaSeq 6000 using PE150 sequencing. For reads assembly, we used Spades (v3.13.0) (38) (http://cab.spbu.ru/software/spades/) with the “–meta” parameters. As the sample contained many unknown genes, we predicted gene sequences using two methods—the Prokka software (version 1.11) and phast online prediction. We selected the optimal method between the two. In most cases, there was little difference between these two methods, but phast software was able to provide more specific gene annotation results. Additionally, we compared the predicted protein sequences with the NCBI NR database using the blastp algorithm (diamond version 0.9.30, with e < 1e-5 parameters) to identify the possible classification and gene annotation of these proteins (39). BLASTP (http://www.ncbi.nlm.nih.gov/BLAST/) analyses were used to identify putative homologies with predicted phage proteins. Comparative genomics analysis of related phages was carried out using Easyfig 2.3 (40). The phylogenetic tree of the full-length genome was constructed using the Neighbor-Joining method of MEGA11 software. The bootstrap values were calculated from 1,000 trees and values only greater than 70 were displayed.

### Studies of bacteria-phage interactions.

Phages were mixed with periodate or proteinase K treated Y. pestis cells as described previously (12). The cells after treatment were washed to eliminate the impact of residual proteinase K. Then the suspensions were incubated at room temperature for 5 min and centrifuged at 16,000 g for 3 min. Finally, the titer of free phages in the supernatant (residual PFU%) was determined. BHI (Brain-Heart Infusion Broth) was used as a nonadsorbing control in each assay, and the phage titer in the control supernatant was set to 100%. The *waaA* gene of the LPS core biosynthetic pathway of Y. pestis was deleted by replacing with kanamycin resistance cassette (6). The recombinant plasmid pACYC184 containing the *waaA* gene-coding region was subsequently introduced into the mutants, yielding the complemented mutant strain, 201C-*waaA* (6).

### Identification of the temperature dependent infection.

Y. pestis was cultured at 26°C and 37°C. Aliquots of each were mixed with the phages and 3 mL of molten top soft nutrient agar (0.7% agar), and then the mixture was overlaid on the solidified base nutrient agar (1.5% agar). Following incubation at 26°C and 37°C, presence of phage plaques was checked. Adsorption assay using Y. pestis grown at different temperatures was conducted, phages were mixed with Y. pestis grown at 26°C and 37°C as described previously (12). After centrifugation, the titer of free phages in the supernatant (residual PFU%) was determined. The purpose of this experiment is to evaluate the presence of bacteriophage receptors on the surface of bacteria cultured at different temperatures. To identify lysogens, Y. pestis was infected with the phage at 26°C for 12 h, and the bacteria were plated on LB agar and incubated at 26°C. The colonies were randomly picked and tested for phage DNA by PCR. The PCR primers used in this study were designed using Primer-BLAST targeting the capsid gene (F: CCGTGGGTATTTCCCGTGAT; R: TATTGCCATCCGCGAACAGT) of the Mu-like phages. PCRs were carried out in a T100 thermocycler (Bio-Rad) with the following cycling conditions: 94°C for 5 min, 30 cycles of 94°C for 30 s, 65°C for 30 s, and 72°C for 40 s, followed by a final extension of 5 min at 72°C. PCR products were analyzed on a 1% (wt/vol) agarose gel. The PCR positive colonies were cultured in 5 mL LB at 37°C and then centrifuged at 16,000 *g*, 3 min at 4°C; the supernatant was used for double-layer agar assay.

### Data availability.

The nucleotide sequence of vB_YpM_3, vB_YpM_5, vB_YpM_6, and vB_YpM_23 reported in this article has been deposited in the GenBank database as accession number MT374852, MT374853, MT374854, and MT374856.
