# Supplementary figures and images for: Characterization of Mu-Like Yersinia Phages Exhibiting Temperature Dependent Infection
Source: Microbiol Spectr. 2023 Jul 19;11(4):e00203-23. doi: 10.1128/spectrum.00203-23 (PMC10434027; doi:10.1128/spectrum.00203-23)

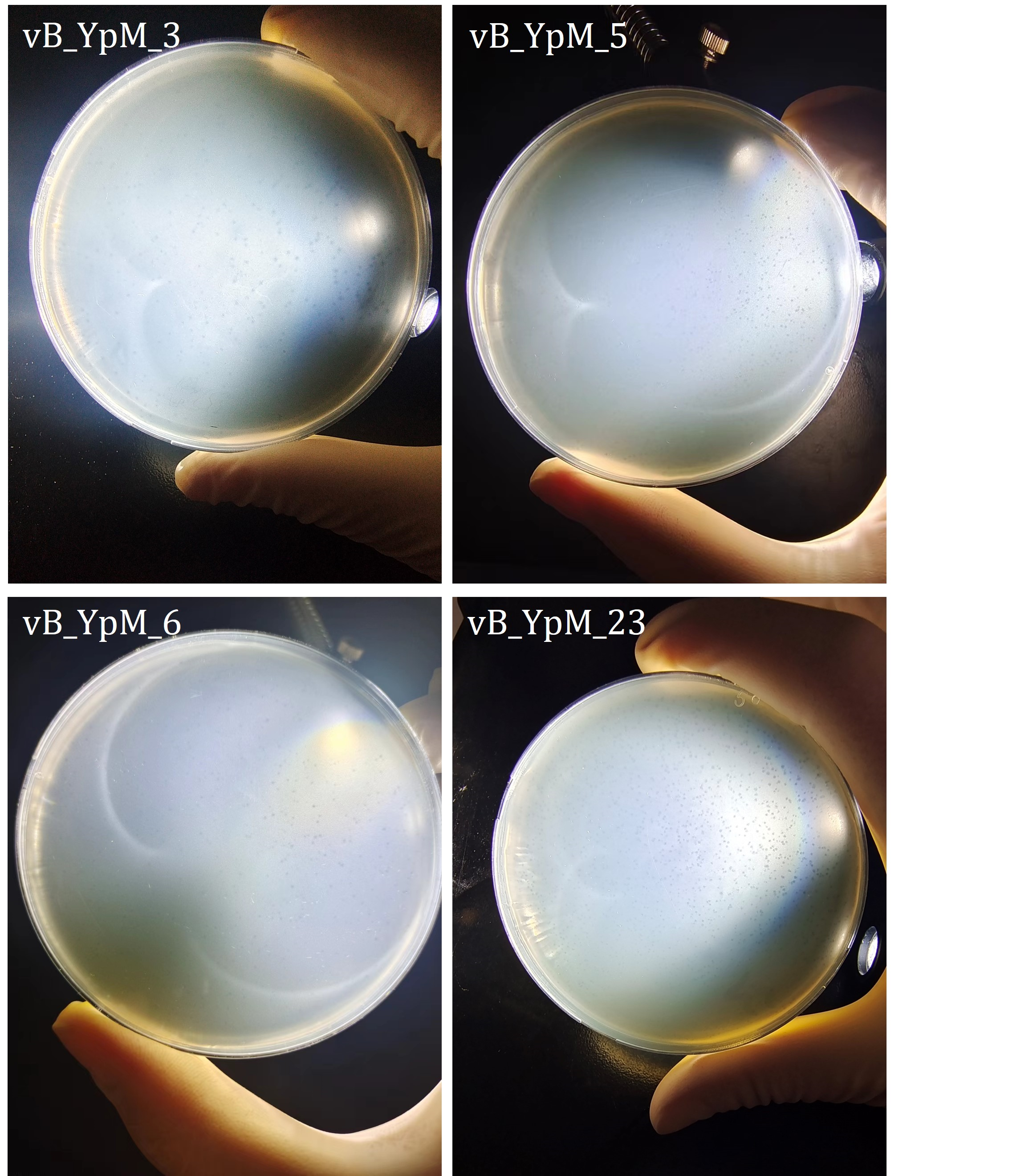

Supplement: Supplemental file 1 — Fig. S1. Download spectrum.00203-23-s0001.tif, TIF file, 6.0 MB [file spectrum.00203-23-s0001.tif]

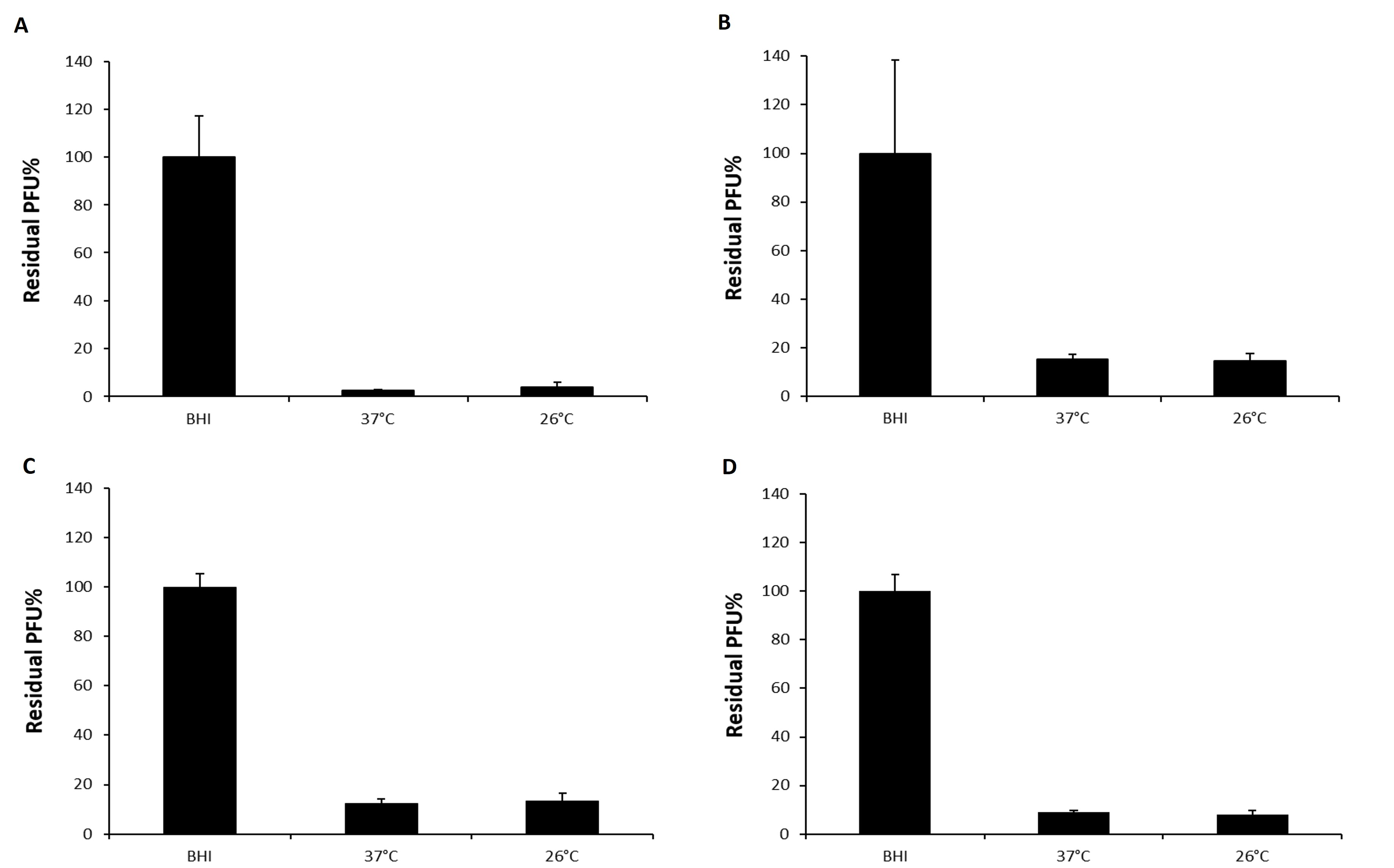

Supplement: Supplemental file 2 — Fig. S2. Download spectrum.00203-23-s0002.tif, TIF file, 1.0 MB [file spectrum.00203-23-s0002.tif]

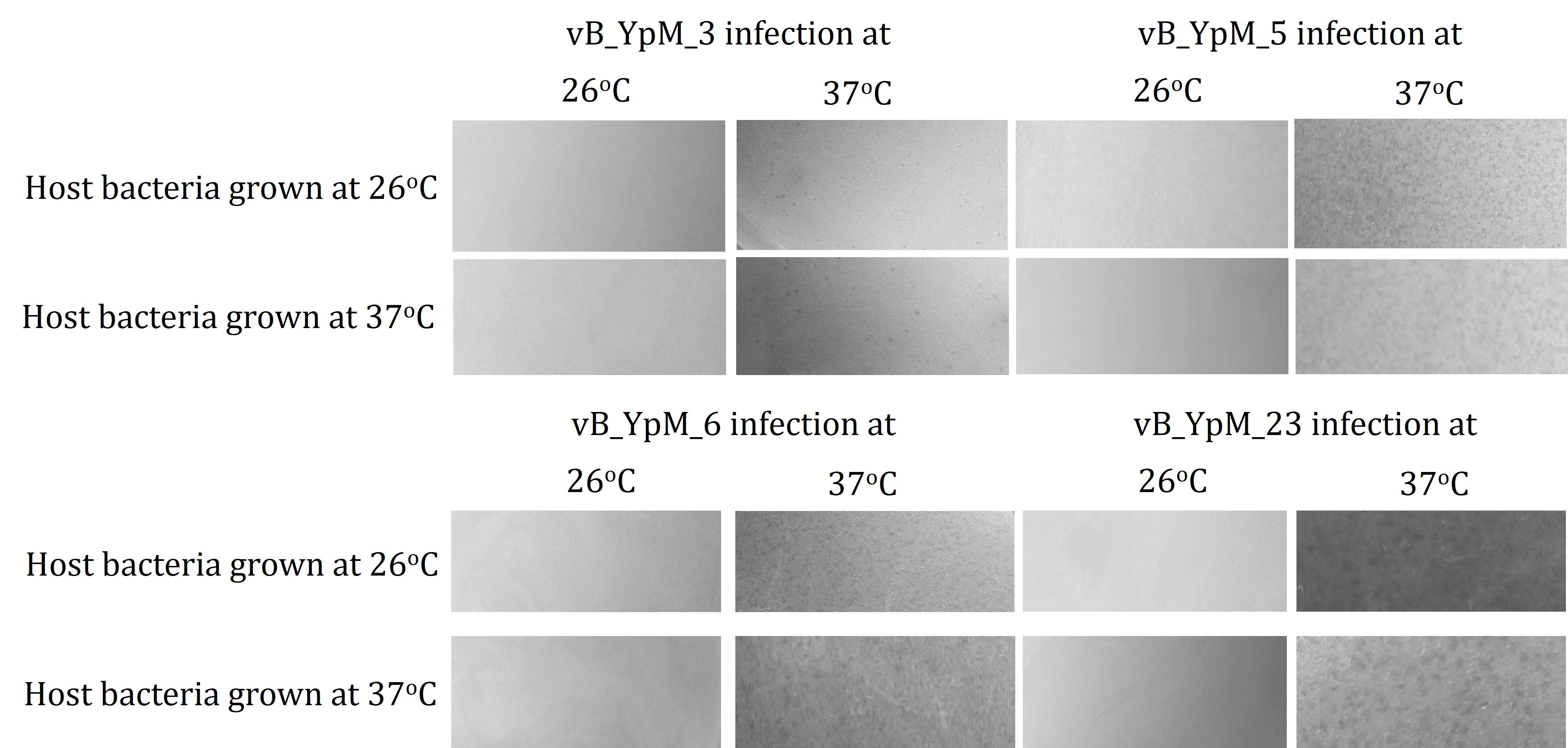

Supplement: Supplemental file 3 — Fig. S3. Download spectrum.00203-23-s0003.tif, TIF file, 3.3 MB [file spectrum.00203-23-s0003.tif]

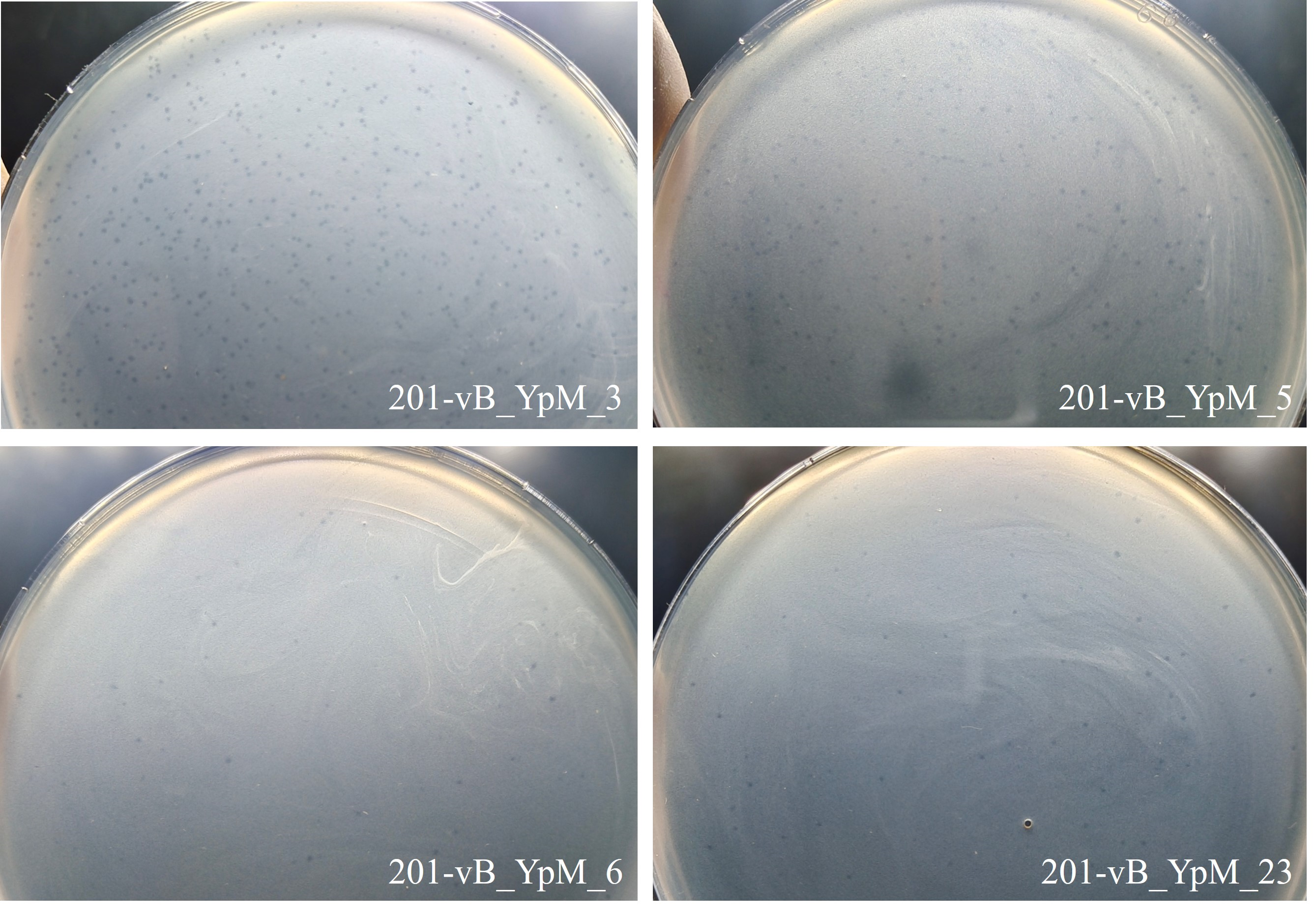

Supplement: Supplemental file 4 — Fig. S4. Download spectrum.00203-23-s0004.tif, TIF file, 5.1 MB [file spectrum.00203-23-s0004.tif]

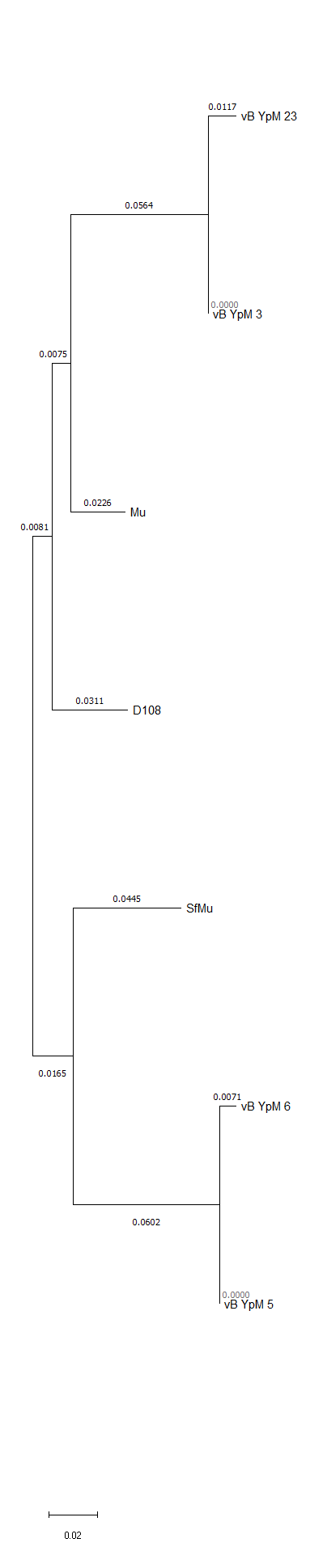

Supplement: Supplemental file 5 — Fig. S5. Download spectrum.00203-23-s0005.tif, TIF file, 3.0 MB [file spectrum.00203-23-s0005.tif]
