# Supplementary material for: Characterization of Mu-Like Yersinia Phages Exhibiting Temperature Dependent Infection
Source: Microbiol Spectr. 2023 Jul 19;11(4):e00203-23. doi: 10.1128/spectrum.00203-23 (PMC10434027; doi:10.1128/spectrum.00203-23)
Supplement: Supplemental file 6 — Legends for supplemental figures. Download spectrum.00203-23-s0006.docx, DOCX file, 0.01 MB [file spectrum.00203-23-s0006.docx]

Supplementary figure 1. Plaques of the phages (vB_YpM_3, vB_YpM_5, vB_YpM_6 and vB_YpM_23) on *Y. pestis* strain 201.

Supplementary figure 2. Adsorption assay using *Y. pestis* grown at 26 °C and 37 °C, shown as residual PFU percentages. The phages can recognize and adsorb *Y. pestis* strain 201 from different culture temperatures, confirming that phage receptors are normally expressed at different temperatures and can be recognized by phages. Significance was determined by independent-sample T test. *, *P* < 0.05, **, *P* <0.01, ***, *P* <0.001. vB_YpM_3 (A), vB_YpM_5 (B), vB_YpM_6 (C) and vB_YpM_23 (D). BHI: the non-adsorbing control; 37 °C: *Y. pestis* strain 201 grown at 37 °C; 26 °C: *Y. pestis* strain 201 grown at 26 °C. Each data point is a mean of three different replications of the same experiment.

Supplementary figure 3. The impact of temperature on susceptibility of *Y. pestis* to the phages. when the temperature of phage infection was 26°C, the phages could not lysis *Y. pestis*, regardless of the growth temperature of *Y. pestis* used for double-layer agar assay is 26 °C or 37 °C, which proved that phage lysis was independent of the growth temperature of the host bacteria. Bacteria grown at both 26°C and 37°C were susceptible only when the temperature of phage infection was 37°C. Representative images from at least three independent experiments are shown.

Supplementary figure 4. After lysogens at 26 ° C was transferred to 37 ° C for culture, lysogenic phages transformed into lytic bacteriophages, they lysis the host bacteria and the progeny phages were released. The bacteriophage particles were dissociated in the supernatant after centrifugation, and the supernatant were determined by double-layer agar assay, on which many plaques could be clearly seen. 201-vB_YpM_3: Lysogenic *Y. pests* strain 201 carrying vB_YpM_3; 201-vB_YpM_5: Lysogenic *Y. pests* strain 201 carrying vB_YpM_5; 201-vB_YpM_6: Lysogenic *Y. pests* strain 201 carrying vB_YpM_6; 201-vB_YpM_23: Lysogenic *Y. pests* strain 201 carrying vB_YpM_23.

Supplementary figure 5. Phylogenetic tree of selected phages based on based on the whole genome. Evolutionary analyses were conducted in MEGA11. The evolutionary history was inferred using the Neighbor-Joining method.
